# Interpersonal behaviors in sports context: Spanish adaptation and measurement invariance

**DOI:** 10.3389/fpsyg.2024.1343063

**Published:** 2024-02-27

**Authors:** Heriberto Antonio Pineda-Espejel, Raquel Morquecho-Sánchez, Verónica Morales-Sánchez

**Affiliations:** ^1^Facultad de Deportes, Universidad Autónoma de Baja California, Mexicali, Mexico; ^2^Facultad de Organización Deportiva, Autonomous University of Nuevo León, San Nicolás de los Garza, Mexico; ^3^Facultad de Psicología, Universidad de Málaga, Málaga, Andalusia, Spain

**Keywords:** multi-group analysis, interpersonal behavior, psychological needs, sport, validating a measure

## Abstract

**Introduction:**

The interpersonal behavior questionnaire (IBQ) is an instrument that measures support and thwarting interpersonal behaviors based on the self-determination theory (SDT). The aim of this work was to adapt the IBQ to the Spanish spoken in Mexico and to examine its psychometric properties (structural validity, discriminant validity, composite reliability, factorial invariance, and nomological validity) in a sample of athletes.

**Methods:**

For this purpose, 472 athletes (average age 17.15 years; SD = 1.47) completed a question booklet.

**Results and discussion:**

Confirmatory factor analysis supported the structure of six related factors, three factors of behaviors that support autonomy, competence, and relatedness, and three factors of behaviors that thwarting them. The internal consistency of each factor was also supported, as well as the average variance extracted. However, the discriminant validity between the factors of competence and relatedness in their dimensions of support, on the one hand, and thwarting, on the other, is questioned. Factorial invariance was confirmed across gender (men and women) and sport type (individual and team). Nomological validity is in accordance with theory and empirical literature. More studies of the IBQ in sport are necessary to see if these results are a fortuitous product or if they manifest themselves consistently.

## Introduction

A factor that could explain how and why the people we interact with have an impact on the quality of our psychosocial experiences, is the way they influence our psychological needs (Sheldon, 2011). In sports, the coach could be considered the main person for athletes, thus playing an important role in the satisfaction of psychological needs (Ryan and Deci, 2007) or in the frustration of them, since the coach is the one who sets goals, supervises training, models attitudes, creates strategies, handles competition situations, and many other activities where the athletes’ pressure to demonstrate a certain level of performance can sometimes make the coach treat his/her athletes inadequately (Mageau and Vallerand, 2003).

Self-determination theory (SDT; Ryan and Deci, 2000) proffers that people have three basic psychological needs (BPN), autonomy (i.e., acting in line with their own interests and values), competence (i.e., experiencing a sense of effectiveness), and relatedness (i.e., experiencing a sense of connectedness with other people). These are innate, are present in all stages of development, and are universal throughout all cultures (Deci and Ryan, 2002). The satisfaction of these BPN by the social context provides people the psychological nutrients necessary for a person’s optimal functioning, which further impacts the quality of motivation, promoting positive or negative consequences on wellbeing, because based on this theory, the conduct’s internalization process requires the support and satisfaction of the three BPN (Deci and Ryan, 2002).

The social context includes both the sport’s structure (i.e., competitive environment), as well as the people within the sport (i.e., coach). The people of the social context can support or thwart the individual perception of the BPN depending on their interpersonal styles, a construct employed to characterize the way people interact among themselves during social exchanges (Deci and Ryan, 2002). SDT proffers that interpersonal conducts of coaches play an important role in the experiences of athletes based on the degree in which these conducts support or thwart the BPN (Deci and Ryan, 1985); for example, when the people of the sport context exhibit interpersonal conducts that support autonomy, then they will promote BPN satisfaction in the athlete (Deci and Ryan, 1985).

Additional to supporting the autonomy, SDT proposes the existence of three interpersonal styles that support BPN, and three interpersonal styles that thwart BPN. The conducts that support autonomy (AS) are described as those that provide explanation for the tasks, recognize the perspectives and viewpoints of others, and bring opportunities for initiative in tasks (Mageau and Vallerand, 2003; Mageau et al., 2015). The conducts that thwart autonomy (AT), or controlling conducts, include using conditional rewards, using intimidating or coercive language, asking for tasks without a justification, and making use of an excessive personal control (Bartholomew et al., 2009). The conducts that support competence (CS) include the use of realistic expectations, the importance of learning, providing positive feedback, recognizing improvement, believing in people’s ability to reach goals, and encouraging them to improve their skills (Sheldon and Filak, 2008). The conducts that thwart competence (CT) consist in emphasizing on other’s mistakes, discouraging people from trying to carry out difficult tasks, sending the message that somebody is incompetent, and doubting others’ ability to improve (Sheldon and Filak, 2008; Reeve, 2015). The conducts that support relatedness (RS) include showing that you understand, support and care for others, displaying warmth (Jones et al., 2004), and showing an interest in the activities of others. Lastly, the conducts that thwart relatedness (RT) include being distant from others, excluding persons from the group or activities, not listening, and not being available when necessary (Sheldon and Filak, 2008; Rocchi et al., 2017).

These constructs are not opposites, and people can engage in both interpersonal conducts of supporting and thwarting (Vansteenkiste and Ryan, 2013), in other words, they can coexist simultaneously within one context (Vansteenkiste et al., 2020).

Of these six interpersonal theorized styles, AS is the one that has received the most attention, partly because the need for autonomy is at both the most exclusive of the SDT and the most controversial. Thus, to this day, research has focused mainly on AS, supporting that the latter predicts the satisfaction of the BPN in sports (Adie et al., 2008; Balaguer et al., 2008; Álvarez et al., 2013; Morillo et al., 2018; Heredia-León et al., 2023). The controlling style (AT) has been studied to a lesser degree, even though it is necessary to measure supporting as well as thwarting conducts, since the absence of support does not imply the presence of thwarting conducts (Sheldon, 2011; Vansteenkiste et al., 2020). For this reason, some studies have simultaneously analyzed AS and AT, where AS leads to greater autonomous motivation, positive affects, and self-confidence; while AT leads to more controlled motivation, amotivation and negative affects (Isoard-Gautheur et al., 2012; Haerens et al., 2015; Behzadnia et al., 2018; Pineda-Espejel et al., 2020).

The aforementioned empirical evidence suggests that the socializing agents that support autonomy also tend to support the competence and relatedness needs. Thus, when coaches support autonomy, they often also support other needs of their athletes (i.e., competence and relatedness). In general, AS has been seen as the promoter of satisfaction of autonomy and relatedness, nevertheless, the question emerges regarding to what happens to the other interpersonal conducts proposed by SDT (i.e., CS, RS, CT, RT), or if supporting autonomy is enough to favor the motivational processes.

In the sports context, little is known about the other four interpersonal styles proposed by SDT, and their influence in the satisfaction and frustration of the BPN of athletes (Rocchi et al., 2017). Maybe it’s due to the lack of valid instruments to measure the six interpersonal styles in the aforementioned context.

It’s important to measure the perception of the three types of interpersonal conducts that support the needs, and the three types of interpersonal conducts that thwart the same, to be able to know how coaches can nurture the psychological needs of their athletes. That is why progress has been made in the efforts to measure each one of the six interpersonal styles that support/thwart the BPN, for example, the observational guide of Haerens et al. (2013) within the context of physical education. One weakness is that the observation analyzes the frequency of conducts but not whether those conducts are perceived as being supporting or thwarting. On the other hand, some instruments only measure autonomy support, ignoring the role of competence and relatedness support, while others measure the support of the three BPN, and others measure the thwarting of BPN, instead of measuring them together as a whole. For this reason, in some cases various instruments are used to measure on one hand the support of BPN, and on the other the thwarting of the same, which may lead to conceptual differences among the different tools, as well as inconsistencies in measurements (Rodrigues et al., 2019).

In light of the need to measure the degree in which interpersonal conducts are either supporting or thwarting, focusing on conducts and not feelings, in other words, the current perceptions of the conducts, the Interpersonal Behaviors Questionnaire applied to the context of sports (IBQ in sport; Rocchi et al., 2016). It consists of a self-report that assesses the athlete’s perception of the coach’s interpersonal behaviors that support or thwart the athlete’s basic psychological needs. Furthermore, the IBQ has been applied to the context of physical exercise with users of gymnasiums in the Portuguese language (Rodrigues et al., 2019), it has been translated to Spanish and applied to the context of physical education by Burgueño and Medina-Casaubón (2021), and translated into Romanian applying it to the sports context (Alexe et al., 2023). These three versions have demonstrated adequate structural validity, adequate reliability, invariance across sex, as well as nomological validity.

Rocchi et al. (2017) mentioned that in order to confirm IBQ’s universality and applicability in different cultures, it is necessary to analyze the instrument in different contexts and populations, as well as replicating the invariance in other groups with different characteristics. Therefore, validation in a sample of Mexican athletes, as well as evidence of invariance increases the scientific evidence contributing to development of knowledge about the universality of the interpersonal behaviors. Additionally, SDT proffers that the three types of interpersonal conducts are essential, which is why it is important to connect them with the three types of BPN to determine how each dimension of the interpersonal conducts are conceptually different from each other.

Since few Spanish-language instruments simultaneously examine the six dimensions of interpersonal behaviors in the sport context, not much is known about the role of the magnitude of behaviors that support/thwart competition and relatedness, and their effects they may have on motivational experiences in sport. Therefore, this study aims to adapt the IBQ to the Spanish spoken in Mexico and to examine its psychometric properties (factor structure, reliability, factor invariance-men vs. women, team vs. individual sport, adolescents vs. young adults-) in a sample of athletes, and then to examine its nomological or criterion validity by relating the subscales to BPN satisfaction and frustration.

## Methods

### Participants

The information was gathered during the first 2 weeks of the 2022 CONADE’s National Games, National stage, based in Mexicali, México. The selection of the sample was non-probabilistic by convenience, depending on the sports competition and the athletes that could be reached. The sample size was obtained with the ratio of estimated parameters between 10:1 (Tabachnick and Fidell, 2012). A total of 472 federated athletes and national competition level (250 men, 218 women, the rest of the participants preferred not to reveal their gender) between the chronological ages of 13 and 22 years old (M = 17.15; SD = 2.81) participated. Information reported an average frequency of training of 4.68 days a week (SD = 1.42), for 3.32 h per day on average (SD = 1.41). They had an average training experience in their sport of 5.74 years (SD = 3.00). The sports accessed for data collection were field hockey (*n* = 192), fencing (*n* = 148), modern pentathlon (*n* = 50), bowling (*n* = 34), soccer (*n* = 24) and wrestling (*n* = 24).

### Instruments

The Interpersonal Behaviors Questionnaire Spanish versión (Burgueño and Medina-Casaubón, 2021) was used. This instrument measures the athletes’ perception about the interpersonal behaviors of their coaches. It consists of 24 items which measure six BPN interpersonal supporting and thuwarting behaviors proposed by the SDT, which make up the six subscales of the instrument (AS, AT, CS, CT, RS, RT). It is answered with a seven-point Likert scale ranging from 1 (*completely disagree*) to 7 (*completely agree*).

To measure the athletes’ BPN, both in its satisfaction and its frustration, the Spanish version of the Basic Psychological Need Satisfaction and Frustration Scale was used (BPNSFS; Pineda-Espejel et al., 2023). It consists of 24 items clustered in six factors according to the satisfaction and the frustration of the competence needs (e.g., “I feel capable about what I do”/“I feel insecure about my skills”), autonomy needs (e.g., “I feel like I do what I really want”/“I feel under pressure to do a lot of things”), and relatedness needs (e.g., “I feel affection for the people I spend time with”/“I feel excluded from the group I want to belong to”). Each factor is made up of four items, which are answered with a five-point Likert scale ranging from 1 (*completely false*) to 5 (*completely true*).

### Procediment

The study received the ethical approval of the university of the first author (UABC-1152). The Spanish version of the IBQ had a contextual linguistic adaptation to the Spanish spoken in Mexico, so grammatical adjustments were made to make it more appropriate to the context. After having the final of the instrument, the first contact was with the organizing institution of the event (Institute of Sport and Physical Culture of Baja California) to obtain its authorization for its application during the event. Subsequently, sports delegates and coaches were informed about this study during the technical board of each sport. We went to the competition sports facilities to request the support of the coaches so that their athletes could answer the questionnaires. Data collection was carried out in the training session prior to the competition. Athletes of legal age were informed in writing and gave their consent to participate; in the case of minors, consent was provided by the coach, since most dads or moms were not present at that time. All participants were treated in accordance with the ethical guidelines of the American Psychological Association, so that the confidentiality and anonymity of the responses are guaranteed.

### Data analysis

First, it was found that the database did not have atypical responses or lost values. Descriptive and univariate normality statistics of the items were analyzed with the SPSS 23 program. Then, to examine the factor structure, confirmatory factor analysis (CFA) was carried out with the Mplus program, and the maximum plausibility estimation method (ML), where age was not included as a covariate. The model evaluation was carried out using absolute and incremental adjustment indices. These included the RMSEA and its 90% confidence interval (90 CI), RSMR, TLI, and CFI. Values equal to or less than 0.08 for RMSEA indicate good fit (Hair et al., 2019), with values equal to or less than 0.10 for the upper limit of 90 IQ (Byrne, 2016). For the RMSR, values equal to or less than 0.08 reflect an optimal setting when used in combination with other indices (i.e., RMSEA) more than when used in isolation (Hu and Bentler, 1999); and for CFI and TLI values ≥0.90 indicate acceptable adjustment (Hu and Bentler, 1999).

The equivalence of the instrument with a multigroup CFA was also examined to test factorial invariance through gender, age group (teenagers or young adults) and type of sport (team or individual). Differences no >0.01 for CFI indicate irrelevant practical differences (Cheung and Rensvold, 2002); the same for RMSEA when the increases are <0.015.

Additionally, a reliability analysis was carried out with McDonald’s omega coefficient, where values >0.70 show good reliability (Hair et al., 2019), and the average extracted variance (AVE) was tested, where values >0.50 indicate good fit (Hair et al., 2019). Finally, the validity of the criterion was analyzed through the relationship with the satisfaction and frustration of each of the BPNs.

## Results

### Factorial analysis confirmatory

CFA confirmed the measurement model formed by a structure of six related factors, since data offers a good representation of reality: *χ*^2^ (237) = 578.85, *p* < 0.001; RMSEA = 0.06 (90% CI = 0.05–0.07); CFI = 0.92; TLI = 0.91; SRMR = 0.07. All items saturated over 0.40, with a significance of *p* < 0.01 (Table 1). However, the phi correlation matrix (Table 2) showed high correlations between the latent factors of CS and RS (phi = 0.94) on the one hand, and between CT and RT (phi = 0.95) on the other, suggesting a lack of discrimination between these factors.

**Table 1:** Descriptive statistics, normality, and factorial weights of the items that make up the IBQ in sport.

Item	λ	ε	R^2^	*M*	*SD*	Asymmetry	Kurtosis
*Interpersonal behavior of autonomy support (AS)*							
1 Gives me the freedom to make my own choices (Me da libertad de tomar mis propias decisiones)	0.68	0.19	0.47	5.64	1.56	−0.99	0.08
7 Supports my decisions (Apoya mis decisiones)	0.78	0.21	0.61	5.81	1.42	−1.27	2.45
13 Supports the choices that I make for myself (Apoya las elecciones que hago)	0.80	0.17	0.65	5.62	1.51	−1.02	0.35
19 Encourages me to make my own decisions (Me anima a tomar mis propias decisiones)	0.76	0.18	0.59	5.59	1.56	−1.10	0.63
*Interpersonal behavior of competence support (CS)*							
2 Encourages me to improve my skills (Me anima a mejorar mis habilidades)	0.80	0.13	0.64	6.10	1.32	−1.07	2.64
8 Provides valuable feedback (Me proporciona correcciones útiles)	0.66	0.16	0.43	6.09	1.40	−1.70	2.45
14 Acknowledges my ability to achieve my goal (Reconoce mi habilidad para lograr mis metas)	0.81	0.12	0.66	5.99	1.35	−1.33	1.18
20 Tells me that I can accomplish things (Me dice que puedo lograr las cosas)	0.83	0.10	0.70	6.05	1.44	−1.70	2.48
*Interpersonal behavior of relatedness support (RS)*							
3 Is interested in what I do (Se interesa por lo que hago)	0.87	0.08	0.77	5.98	1.44	−1.48	1.58
9 Takes the time to get to know me (Se toma el tiempo para saber sobre mí)	0.91	0.08	0.83	5.59	1.68	−1.15	0.48
15 Honestly enjoy spending time with me (Realmente le gusta pasar tiempo conmigo)	0.85	0.08	0.72	5.25	1.55	−0.72	−0.01
21 Relates to me (Se relaciona conmigo)	0.84	0.08	0.71	5.51	1.68	−0.94	0.02
*Interpersonal behavior of autonomy thwarting (AT)*							
4 Pressures me to do things their way (Me presiona para hacer las cosas a su manera)	0.46	0.05	0.22	3.48	1.94	0.28	−1.11
10 Imposes their opinions on me (Me impone sus ideas)	0.55	0.06	0.30	4.22	2.11	−0.18	−1.28
16 Pressures me to adopt certain behaviors (Me presiona para comportarme de cierta forma)	0.67	0.04	0.45	3.34	2.05	0.33	−1.19
22 Limits my choices (Limita mis decisiones)	0.69	0.03	0.48	2.77	1.99	0.82	−0.62
*Interpersonal behavior of competence thwarting (CT)*							
5 Points out that I will likely fail (Me dice que seguramente fallaré)	0.84	0.04	0.70	2.28	1.95	1.26	0.11
11 Sends me the message that I am incompetent (Me envía mensajes de que soy torpe)	0.90	0.04	0.82	2.24	2.00	1.33	0.21
17 Doubts my capacity to improve (Duda de mis capacidades para mejorar)	0.86	0.05	0.74	2.50	1.99	1.01	−0.37
23 Questions my ability to overcome challenges (Cuestiona mis habilidades para superar algún desafío)	0.79	0.05	0.63	2.58	1.91	0.95	−0.38
*Interpersonal behavior of relatedness thwarting (RT)*							
6 Does not comfort me when I am feeling low (No me consuela cuando me siento mal)	0.66	0.06	0.44	3.18	2.20	0.45	−1.31
12 Is distant when we spend time together (Es distante cuando pasamos tiempo juntos)	0.90	0.05	0.81	2.84	2.95	0.86	−0.52
18 Does not connect with me (No conecta conmigo)	0.86	0.05	0.75	2.48	1.79	0.82	−0.73
24 Does not care about me (No se preocupa por mí)	0.82	0.05	0.68	2.31	2.01	1.20	−0.01

λ, factorial weight; ε, standard error; *M*, average; *SD*, standard deviation.

Additionally, the correlation matrix (Table 2) confirmed positive associations between each of the three factors measuring the perception of BPN-supportive interpersonal behaviors, on the one hand, and between each of the three factors measuring the perception of BPN-frustrating interpersonal behaviors, on the other hand.

**Table 2:** Matrix of phi correlations between the latent factors of IBQ in sport, AVE, and reliability (diagonal).

Interpersonal behavior of …	AS	CS	RS	AT	CT	RT
Autonomy support (AS)	0.92					
Competence support (CS)	0.30^**^	0.96				
Relatedness support (RS)	0.28^**^	0.94^**^	0.97			
Autonomy thwarting (AT)	−0.49^**^	0.23^**^	0.31^**^	0.96		
Competence thwarting (CT)	−0.07	−0.81^**^	−0.72^**^	0.12	0.98	
Relatedness thwarting (RT)	−0.12^*^	−0.82^**^	−0.84^**^	0.03	0.95^**^	0.98
AVE	0.57	0.60	0.75	0.36	0.72	0.66

^**^p < 0.01; ^*^p < 0.05.

Table 2 shows that, in terms of reliability, the omegas coefficients were satisfactory, exceeding the criterion of 0.70 (McDonald omega range of 0.97–0.99). While the AVE indicated that the constructs explain more than half of the variance of all the indicators that compose them, except for the AT factor.

### Factorial invariance

To analyze factorial invariance across gender, age group (adolescents vs. young adults), an sport type (individual vs. team) a series of multisample confirmatory factor analyses were performed (Table 3). In the gender case, these indicated that, based on the differences in RMSEA the six related factor structure of the instrument is invariant between males and females athletes, since comparisons of this index between the nested models with restrictions confirmed equivalences in all four models. That is, first the levels of configural invariance (M1) were confirmed. Then, by comparing M2 with the previous model, the measurement invariance could be confirmed. When comparing M3 with M1, scalar equivalence (strong invariance) was confirmed. Finally, the comparison of M4 with M1 could confirm the residual equivalence (strict invariance) (Byrne, 2016). In the age groups, the results rejected the ivariance hypothesis at all levels between adolescents and youg adults. Finally, the results supported the strict invariance across the sport type, guiven that factorial, covariance and error measurement model equivalence was confirmed.

**Table 3:** Goodness-of-fit rates for each of the models tested on the factorial invariance of the IBQ in sport between men and women.

Model	*χ*^2^ (d.f.)	RMSEA	CFI	ΔRMSEA	ΔCFI
*Across gender*					
M1_ configural invariance	888.69 (474)^*^	0.072	0.940		
M2_ metric invariance	903.49 (492)^*^	0.075	0.920	0.003	0.020
M3_ intercept invariance	968.98 (516)^*^	0.077	0.900	0.005	0.040
M4_ invariance of intercepts and errors	983.56 (540)^*^	0.074	0.900	0.002	0.040
*Across age*					
M1_ configural invariance	945.42 (474)^*^	0.080	0.850		
M2_ metric invariance	987.25 (492)^*^	0.100	0.830	0.020	0.020
M3_ intercept invariance	1044.42 (516)^*^	0.100	0.820	0.030	0.030
M4_ invariance of intercepts and errors	1115.19 (540)^*^	0.100	0.800	0.050	0.030
*Across sport type*					
M1_ configural invariance	1117.73 (474)^*^	0.076	0.909		
M2_ metric invariance	1141.07 (492)^*^	0.066	0.900	0.010	0.009
M3_ intercept invariance	1198.63 (516)^*^	0.065	0.901	0.011	0.008
M4_ invariance of intercepts and errors	1297.05 (540)^*^	0.067	0.899	0.009	0.010

^*^p < 0.001; d.f., degrees of freedom.

### Nomological validity

Regarding the association of the six factors that make up the IBQ in sport with theoretically related constructs, Table 4 shows that each BPN’s supportive interpersonal behavior was positively associated with BPN satisfaction levels and negatively associated with each BPN’s frustration levels. The opposite was true for BPNs’ interpersonal behaviors of frustration.

**Table 4:** Correlation matrix with outcome variables for the IBQ in sport.

	Autonomy satisfaction	Competence satisfaction	Relatedness satisfaction	Autonomy frustration	Competence frustration	Relatedness frustration
AS	0.32^**^	0.23^**^	0.09	−0.16^*^	−0.15^*^	−0.28^**^
CS	0.37^**^	0.26^**^	0.16^*^	−0.15^*^	−0.21^**^	−0.35^**^
RS	0.37^**^	0.28^**^	0.19^**^	−0.05	−0.12	−0.31^**^
AT	−0.23^**^	−0.07	−0.10	0.35^**^	0.27^**^	0.42^**^
CT	−0.33^**^	−0.18^*^	−0.23^**^	0.25^**^	0.35^**^	0.53^**^
RT	−0.36^**^	−0.18^*^	−0.15^*^	0.30^**^	0.38^**^	0.53^**^
*M*	3.81	3.97	4.19	2.84	2.69	2.25
*SD*	0.77	1.16	1.43	1.02	0.97	0.98

*M*, average; *SD*, standard deviation; ^*^p < 0.05; ^**^p < 0.01.

## Discussion

This present study was conducted with the aim of adapting the IBQ to Spanish spoken in Mexico, to examine its psychometric properties in a sample of athletes, and then to examine the nomological or criterion validity by relating the subscales to BPN satisfaction and frustration.

The results support the construct validity of the instrument, since the six latent factor structure is confirmed, in addition, it shows adequate reliability, and it can be said that each construct explains more than half of the variance of the items that compose it, with the exception of the AT factor, however, AVE values slightly below 0.50 can also be considered acceptable (Hair et al., 2019). However, the discriminant validity between latent factors is not satisfactory, since some factors correlate above 0.85 (i.e., CS with RS, y CT with RT), which has happened in other studies of linguistic adaptation and validation in the sports context (Alexe et al., 2023), and of validation to physical education (Burgueño and Medina-Casaubón, 2021) where AS with CS, AS with RS, CS with RS or CT with RT correlated above 0.90; although there are other studies where discriminant validity is confirmed (Rocchi et al., 2017; Rodrigues et al., 2019). This may mean that in the sporting context a coach who performed more CR could also promote more RS. This discrepancy may be due to the fact that across cultures there may be variations in the functional significance or meaning that individuals attribute to a behavior relevant to psychological need.

Regarding nomological validity, the latent factors are associated with satisfaction/frustration for each BPN, as theorized from SDT, and coincides with the validation studies of Rocchi et al. (2017), Rodrigues et al. (2019), and Alexe et al. (2023). This helps to support that the constructs are conceptually distinct from each other. It also suggests that socializing agents capable of nurturing one need often simultaneously support other needs, even if at different intensity, as noted by some authors (Baard et al., 2004; Skinner et al., 2005; Rocchi et al., 2017). In the same way, authority figures who exhibit interpersonal thwart behaviors one need appear to thwarting the other needs to different extents. In this aspect, the latent factors of the measurement instrument represent the constructs theorized in SDT on interpersonal behaviors.

Regarding the multigroup analysis, this supports that the structure of the measurement model is equivalent in the different groups (men vs. women, team vs. individual sport) since the results of the differences in RMSEA results empirically support that the different groups of Mexican athletes interpret the meaning of support and thwart interpersonal behaviors similarly. Although the differences in CFI exceed the suggested limits across age, differences in RMSEA less than 0.015 can support invariance (Chen, 2007). However, it is suggested to take these results with caution, because RMSEA tend to over reject an invariant model when sample size is small (Chen, 2008). These results is added to the evidence of universality and generalizability of the dimensions of interpersonal behaviors related to BPN support and thwarting, what will allow analyzing possible differences in the perception of the six specific dimensions of interpersonal behaviors, as in other studies (Burgueño and Medina-Casaubón, 2021; Alexe et al., 2023). Nevertheless, in the case of age group, the differences in the adjustment indices show that adolescents and young adults interpret the meaning of the items in different ways.

With this self-report at the contextual level, athletes can inform their perceptions of their coach’s interpersonal behaviors. It can be used in the sport context to more accurately measure the role of the six dimensions of supportive and thwarting interpersonal behaviors in predicting BPNs in both satisfaction and frustration, with the understanding that BPN frustration and satisfaction may have different antecedents (Bartholomew et al., 2011). Thereby extending research in sport motivation.

It may then promote future research initiatives that test the complete sequence proposed by Vallerand (1997), at the contextual level, of social factors (i.e., the role of the six types of supportive/thwarting interpersonal behaviors), psychological mediators, motivational types and consequences.

This study is important as the context of sport differs in the degree to which such BPN are generally supported or thwarted (Ryan et al., 2019), particularly coaches’ behaviors may be more or less supportive or thwarting of BPN. From a theoretical perspective, it contributes to the validation and generalization of the construct of interpersonal behaviors in sport in the Mexican population. From a practical perspective, it provides an instrument adapted to the sporting and linguistic context in Mexico, which can be used with adolescent and young athletes of different modalities to reliably evaluate the six types of interpersonal behaviors proposed by the SDT.

However, this study has limitations such as characteristics of the sample, which was not representative of Mexican sport context, and included adolescent and young athletes, which could influence the results due to the comprehension of the items. Therefore, it is suggested to test with other ages, levels of competition and with different Spanish-speaking populations. Studies could also be conducted to learn how perceptions of interpersonal behaviors change over short periods of time. This is a self-report, so it could be triangulated with other more qualitative measures to confirm whether these measures correspond to actual behaviors. On the other hand, the IBQ in sport self was not included in this study, so it is also suggested to validate the instrument with a sample of coaches who report on their own perceptions of their interpersonal behaviors. In general, more studies are needed to continue to test the psychometric properties and extend the validity of the Spanish version of the instrument in the sport context to analyze the possible lack of discriminant validity between the CT-RT and CS-RS subscales, and see if these results are a fortuitous product or if they manifest themselves consistently (Bollen, 1989).

## Conclusion

This study supports the applicability of the Spanish version of the IBQ in Mexican sport, since the factorial structure of the instrument is confirmed, the factors correlate with other theoretically related variables, and it shows to be reliable for measuring athletes’ perceptions of the six types of interpersonal behaviors that their coaches may adopt; although the discriminant validity is questionable. Therefore, this self-report can be applied in the sport context to measure the perception of interpersonal behaviors that athletes report from their coach. This instrument joins those already existing within the literature and measurements within SDT.

## Data availability statement

The raw data supporting the conclusions of this article will be made available by the authors, without undue reservation.

## Ethics statement

The studies involving humans were approved by Universidad Autónoma de Baja California. The studies were conducted in accordance with the local legislation and institutional requirements. Written informed consent for participation in this study was provided by the participants’ legal guardians/next of kin.

## Author contributions

HP-E: Conceptualization, Formal analysis, Investigation, Methodology, Writing – original draft. RM-S: Methodology, Writing – original draft. VM-S: Data curation, Writing – review & editing.
